# Effect of Financially Punished Audit and Feedback in a Pediatric Setting in China, within an Antimicrobial Stewardship Program, and as Part of an International Accreditation Process

**DOI:** 10.3389/fpubh.2016.00099

**Published:** 2016-05-18

**Authors:** Sitang Gong, Xiu Qiu, Yanyan Song, Xin Sun, Yanling He, Yilu Chen, Minqing Li, Rui Luo, Liya He, Qing Wei, Songying Shen, Yu Liu, Lian Zhang, Wei Zhou, Ping Huang, Jianning Mai, Li Liu, Yi Xu, Huiying Liang, Huimin Xia

**Affiliations:** ^1^Department of Gastroenterology, Guangzhou Women and Children’s Medical Center, Guangzhou Medical University, Guangzhou, China; ^2^Division of Birth Cohort Study, Guangzhou Women and Children’s Medical Center, Guangzhou Medical University, Guangzhou, China; ^3^Department of Medical Administration, Guangzhou Women and Children’s Medical Center, Guangzhou Medical University, Guangzhou, China; ^4^Department of Pharmacy, Guangzhou Women and Children’s Medical Center, Guangzhou Medical University, Guangzhou, China; ^5^Department of Pediatrics, Guangzhou Women and Children’s Medical Center, Guangzhou Medical University, Guangzhou, China; ^6^Guangzhou Women and Children’s Medical Center, Institute of Pediatrics, Guangzhou Medical University, Guangzhou, China; ^7^Department of Pediatric Surgery, Guangzhou Women and Children’s Medical Center, Guangzhou Medical University, Guangzhou, China

**Keywords:** prior authorization, audit and feedback, pay for performance, antimicrobial prescription, antimicrobial stewardship, observational study

## Abstract

**Background:**

Prior authorization, audit and feedback, and pay for performance are the three core “active” strategies of antimicrobial stewardship program (ASP), yet little is known about the individual or combined benefits of such programs, particularly in a pediatric setting.

**Objectives:**

The aim of this study was to compare these core ASP strategies and determine the incremental effect of financially punished audit and feedback.

**Methods:**

During the journey to the Joint Commission International accreditation, a tertiary pediatric medical center performed two different hospital-wide stewardship interventions in succession. The first stage without formalized ASPs served as pre-intervention period, January 2011 to April 2011. The ASP used prior authorization alone during the first-intervention period, May 2011 to September 2011. In October 2011, financially punished audit and feedback was introduced, marking the start of the second-intervention period, October 2011 to November 2012. We compared the differences of the change in monthly average use of antibiotics and expenditure on antibiotics before and after the ASP changes by using interrupted time series *via* dynamic regression. The main end points included the proportions of antibiotic prescriptions and expenditure on antibacterial relative to all medications.

**Results:**

Before the second-intervention period, neither the proportion of antibiotic prescriptions nor the proportion of expenditure on antibiotics declined significantly in both ambulatory and inpatient settings. However, after the introduction of financially punished audit and feedback, the proportion of both antibiotic prescriptions (β = −6.269, *P* < 0.001, and reduction = 59.4% for outpatients; β = −1.235, *P* < 0.001, and reduction = 19.8% for inpatients) and expenditure on antibiotics (β = −7.777, *P* < 0.001, and reduction = 46.7% for outpatients; β = −4.933, *P* = 0.001, and reduction = 16.3% for inpatients) dropped immediately.

**Conclusion:**

The combination of more than one core strategies (prior authorization, audit and feedback, and pay for performance) will be more effective than one strategy alone.

## Introduction

According to most standards, the increasing availability of life-saving antibiotics in the developing world is a good thing. But, their widespread availability and inappropriate use have led to the development of multidrug-resistant (MDR) bacterial infections (1, 2). During the past two decades, a dramatic increase in the incidence of nosocomial infections has occurred in children (3). The overuse and misuse of antimicrobial agents are considered key points fueling this situation (4, 5). For instance, antibiotics are prescribed during more than 50% of hospitalizations of children, often unnecessarily (6), which could be even worse in poorer countries than in richer ones, owing in part to a lack of regulation (1).

Antimicrobial stewardship programs (ASPs) are multidisciplinary, hospital-based interventions designed to ensure the appropriate prescription of antibiotics. Despite limited evidence, studies on ASPs have identified several potential strategies, including three core “active” methods: formulary restriction with prior authorization, prospective audit with feedback to prescribers, and pay for performance (7). Prior authorization permits the use of select agents after approval from the ASP team, whereas prospective audit and feedback utilizes post-prescriptive reviews conducted by the ASP to recommend changes in the antibiotic selection, dosing, or duration of therapy (8). Pay for performance programs are intended to strengthen the business case for the improvement of antibiotic usage by rewarding excellence and reversing what have been described as perverse financial incentives (9).

Nevertheless, initial ASP efforts were focused on adult patient populations. In 2010, the Pediatric Infectious Diseases Society (PIDS) formed the Pediatric Committee on Antimicrobial Stewardship with the mission of advancing pediatric ASPs, promoting research in pediatric ASPs, and developing ASPs educational programs. Since then, more concerted efforts for the widespread implementation of formal ASPs in pediatrics have occurred (10). Although promising, current evidences are limited to outpatient services (11) and in high-income countries (12). Thus, concluding that present pediatric ASPs are generally effective in both inpatient and outpatient treatments and both resource-rich and resource-limited settings could be subjected to multiple biases.

Implementation of ASPs to prevent and to control the emergence and spread of antimicrobial-resistant microorganisms is one of the key elements in quality improvement required by the Joint Commission International (JCI). Rational antibacterial use was listed as one of the important patient safety goals in Guangzhou Women and Children’s Medical Center (GWCMC) in 2011–2012, and GWCMC successfully passed the JCI accreditation on December 15, 2012. During the journey to JCI accreditation, GWCMC performed two different hospital-wide stewardship interventions in succession: prior authorization alone and prior authorization + financially punished audit and feedback. The aim of this study was to determine the incremental effect of financially punished audit and feedback, to discuss the effectiveness of such cheap and simple stewardship interventions in the pediatric context, and to provide references for community hospitals and international counterparts.

## Materials and Methods

### Setting and Design

An uncontrolled, observational study focusing on hospital-wide antibacterial use was performed in GWCMC, a 1012-bed tertiary pediatric hospital with 2.65 million outpatient visits, 45,000 Medicare inpatient admissions, and 313,785 inpatient days annually in Guangzhou, China. Approval was obtained from the GWCMC Institutional Review Board.

**Figure 1:**
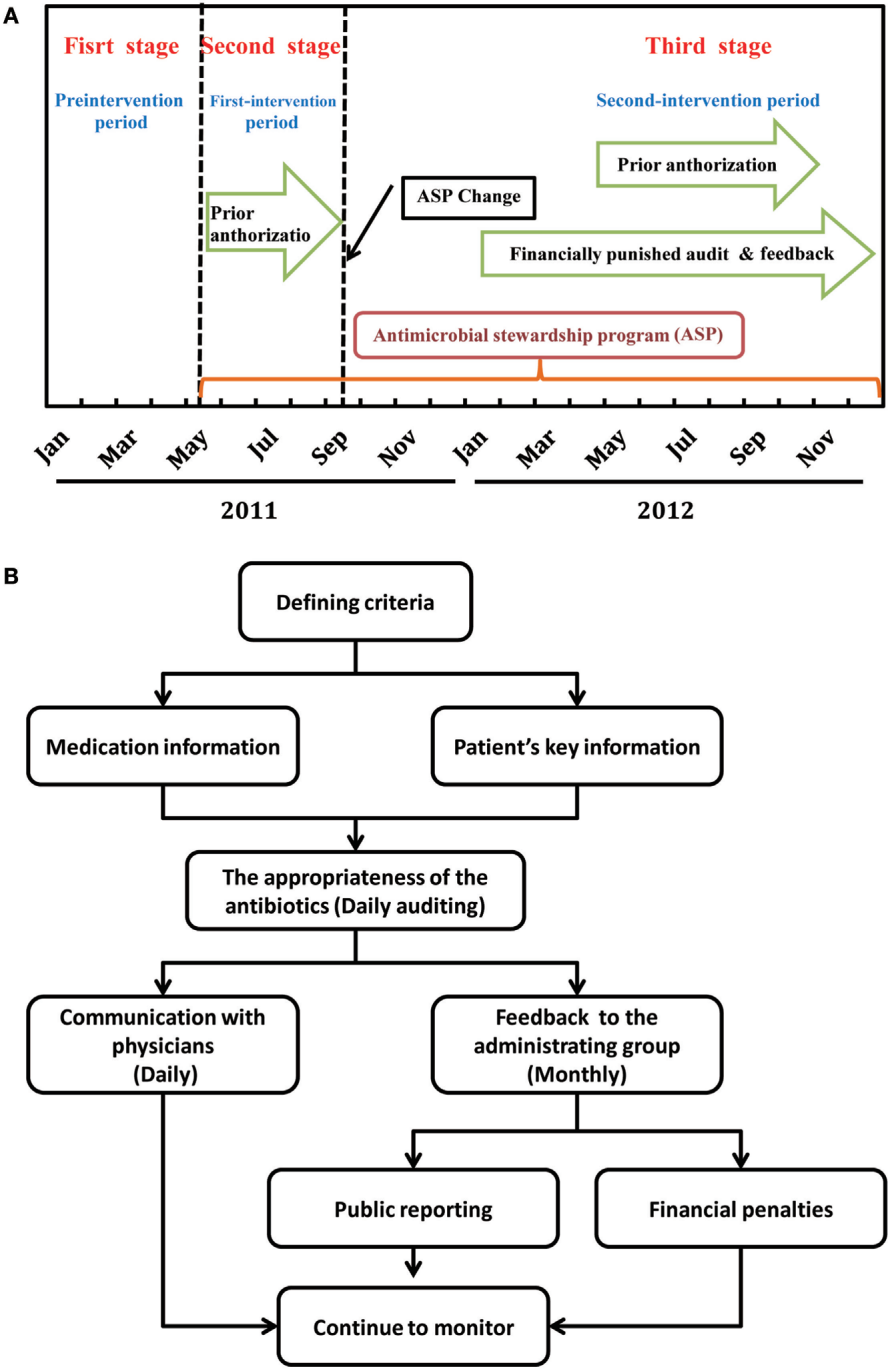
**Timeline of antimicrobial stewardship program (ASP) strategies (A) and flowchart for the intervention of financially punished audit and feedback (B)**.

This was a three-stage study (Figure 1A). The first stage without formalized ASPs served as pre-intervention period and was started from January 1, 2011 to April 30, 2011. In the second stage or the first-intervention period, a formulary restriction with prior authorization according to the campaign protocol launched by the Chinese Ministry of Health in 2011 was implemented between May 1, 2011 and September 30, 2011 (13). In the third stage or the second-intervention period, financially punished audit and feedback was added to the prior authorization since October 1, 2011.

### Antimicrobial Stewardship Programs

#### Establishment of Core Team

Prior to implementation of the programs, a multidisciplinary ASPs committee, including administrating group, supervision group, and implementation group, was established (Figure S1 in Supplementary Material). Administrating group composed of hospital administrators is responsible for providing support to hospital administration and medical staff leadership. Supervision group by pharmacists, a board certified pediatric infectious disease practitioner, information systems experts, and microbiologists bear the main responsibility of the enforcement of antimicrobial restriction policies. The mission of the implementation group by physicians is to fully implement the guidelines.

With reference to the first national guideline on the clinical use of antibacterial medicines published in 2004 (14) and the official document for rational use and standard management of antibiotics issued by Chinese Ministry of Health in 2011 (15), the ASP team developed antimicrobial guidelines for pediatric patients, including choice of antibiotic agent, accuracy of the dose, appropriateness of the route, period of use, and need for combination of antibiotics, and made it available as part of the hospital formulary. The guidelines were made available in written pocket-sized formats and were easily accessible through the hospital Intranet 1 month before starting the ASP. We also developed a set of simple and economical tailor-made indications for the prescription of rational antibiotics (Table S1 in Supplementary Material).

#### Prior Authorization

Antibiotics used in the center were divided into non-restricted (also called “first line”), restricted (“second line”), and special use (“third line”) grades on the basis of their clinical effects and safety. The antibacterial formulary was shown in Table S2 in Supplementary Material. Each grade will match exactly to the corresponding prescribing privileges for physicians, as described previously (16). Briefly, physicians do not need prior approval to prescribe unrestricted antibiotics; senior physicians were allowed to prescribe restricted antibiotics, but access to these antibiotics was strictly controlled for the junior residents and fellows; and all physicians need the special-grade antibiotic expert subcommittee’s approval before they can prescribe special-grade antibiotics.

The antibiotic approval service was staffed by clinical pharmacists and infection control physicians between 8:00 a.m. and 5:00 p.m. on weekdays and by the first- and second-year pharmacist and infection diseases fellows with postgraduate training in anti-infective therapy between 5:00 p.m. and 12:00 p.m. on weekdays and between 8:00 a.m. and 12:00 p.m. on weekends and holidays. Between 00:00 a.m. and 8:00 a.m., restricted and special-grade agents were released pending morning evaluation to prevent delay in appropriate treatment. Of course, if a request was denied, an alternative agent would be suggested.

#### Financially Punished Audit and Feedback

##### Prospective Audit and Feedback

To better utilize the ASP resources, prospective audit with feedback was also introduced in October 2011. In the audit process, an automated report of all patients who had received antimicrobials within the previous 24 h was generated daily from a web-based prescription screening software, except on weekends and holidays. Prospective audits of antimicrobials prescribed during the weekend and holidays were performed on Mondays and the first day after holidays. Critical data elements extracted from the system included type of antimicrobial; dose, interval, and route of administration; patient age, weight, allergies, and renal function; attending physician and admitting service names; and culture results of microbiology for hospitalized patients from any site. The supervision group was responsible for reviewing all of these reports and judging the appropriateness of the use of antibiotics. Subjects were excluded if antibiotic approval had already been authorized by clinical pharmacists and infection control physicians before the call.

Common feedback was performed through direct communication with the attending physician who prescribed the antibiotics, including discontinue the antibiotic; narrow or broaden antimicrobial therapy based on culture and susceptibility data; convert the administration method from parenteral to oral route; increase or decrease the dose; and shorten or lengthen the planned duration of therapy, consolidate to fewer antimicrobials, and obtain an infectious diseases consult. At the end of each month, the data on irrational use of antibiotics were collected and reported to the administrating group by the supervision group. Appropriate evaluation of antibacterial-containing prescriptions was published monthly by the administrating group on the hospital local area network. Figure 1B depicts the workflow of the audit and feedback intervention.

##### Financial Penalties

“Dear doctor” letters were sent from the ASPs team to physicians. Physicians were given the opportunity to present evidence and argument against the results of feedback during a 7-day public notice period. Subsequently, physicians who wrote inappropriate prescriptions would face a hefty fine according to the number of the inappropriate prescriptions (first quartile, second quartile, third quartile, and fourth quartile). The level of fine was correspondingly divided into four levels [first – 500 Chinese Yuan (CNY); second – 700 CNY; third – 900 CNY; and fourth – 1000 CNY]. If a second instance of a fourth-grade error occurs, the responsible physician would face the prescribing privilege revocation and be asked to attend mandatory training on antibiotic use again.

### Measures of Antimicrobial Use

The total number of prescriptions and total number of prescriptions containing antibacterial were derived from the prescription evaluation software embedded in the Hospital Information System (HIS). Data elements for each antibiotic prescriptions include the patients’ demographics (e.g., patient name, identification number, age, diagnosis, allergy history, body weight, body surface area, and clinical laboratory test results), antibiotic usage (generic names, doses, dosing schedules, timing, duration, combinations, and any switchovers to another antibiotic), and cost (antibacterial expenditure and cost of all medications).

### Outcome Measures

The outcome measures included proportion of pediatric antibiotic prescriptions, proportion of prescriptions containing non-restricted antibacterial, proportion of prescriptions containing restricted antibacterial, proportion of prescriptions containing special use grade antibacterial, percentage of monthly antibiotic users of hospitalized children, and proportion of expenditure on antibacterial relative to all medications. Outpatient and inpatient wards were calculated, respectively.

### Statistical Analysis

We determined the differences in the average monthly changes in antibiotic use before and after the ASPs program by using interrupted time series *via* dynamic regression (17). Given the presence of three distinct stages, a model with two change points was used:

$$\begin{array}{c} Y_{t}=\beta_{0}+\beta_{1}\times {\text{time}}_{t}+\beta_{2}\times F_{t}+\beta_{3}\times \text{time after }F+\beta_{4}\times S_{t}+\beta_{5}\times \text{time after }S \end{array}$$

Here, *Y*_t_ is the mean of the indicators used to evaluate antimicrobial use prescription; time_t_ is the number of months at time t from the beginning of the study; “*F*” and “*S*” are the indicators for time t that the “first-intervention (*F*)” was introduced in May (before May = 0 and after May = 1) and the “second-intervention (*S*)” was introduced in October (before October = 0 and after October = 1). Time after “*F*” and “*S*” are the number of months after the first-intervention in May and after the second-intervention in October. In this model, β_0_ estimates the intercept, β_1_ evaluated the baseline trend during pre-intervention period, β_2_ and β_4_ estimate the immediate changes following the first-intervention in May 2011 and the second-intervention in October 2011, β_3_ assesses the change trends of the outcome of interest after the “first-intervention” and before the second-intervention. β_5_ estimates the change trends of the outcome of interest after the second-intervention. In the autoregression model, serial correlation of the error terms was tested by plot of residuals against time and using the Durbin–Watson test. Randomly scattered residuals without a pattern indicate that there is no autocorrelation. Durbin–Watson statistic values close to 2.0 indicate no serious autocorrelation. If the statistic is significant, the models were adjusted by estimating the autocorrelation parameter and including autocorrelated errors by following a second-order autoregressive process. The percentage of changes by the “Second-intervention” was calculated by the formula as follow:

$$\frac{\beta_{4}}{\beta_{0}+\beta_{1}+\beta_{2}+\beta_{3}}\times 100\%.$$

In addition, chi-square test was used for testing the percentage differences between two or more groups. We used *P* < 0.05 as a threshold for statistical significance. All statistical analyses and models were estimated by using SAS 8.1 software (SAS Institute, Cary, NC, USA).

## Results

In total, 29,363,808 medical prescriptions were included in this study; 3,036,274 prescriptions (1,532,336 for outpatients and 1,503,938 for inpatients) were in the pre-intervention period, 4,573,601 prescriptions (2,450,093 for outpatients and 2,123,509 for inpatients) were in the first-intervention period, and 21,753,933 prescriptions (15,499,762 for outpatients and 6,254,171 for inpatients) were in the second-intervention period.

### General Information and Pharmacoeconomic Data on Antibacterial Prescriptions

As shown in Table 1, compared to the pre-intervention stage, significant decreases in the rate of antibiotic prescription were observed both in the first-intervention period and in the second-intervention period for both outpatients (χ^2^ = 7.4 × 10^5^, *P* < 0.001) and inpatients (χ^2^ = 2.0 × 10^4^, *P* < 0.001). During the pre-intervention period, the proportions of expenditure on antibiotics were approximately three times than that of second-intervention period for outpatients (17 vs. 6%) and two times for inpatients (29 vs. 14%). The proportion of hospitalized children who took antibiotics decreased significantly from 40% in the pre-intervention group, to 34% after the implementation of prior authorization alone, and to 23% after adding financially punished audit and feedback (χ^2^ = 3.0 × 10^3^, *P* < 0.001).

**Table 1:** **General information and pharmacoeconomic data on antibacterial prescriptions for outpatients and inpatients by three periods**.

Indicators	Outpatients			Inpatients		
	Pre-intervention	First-intervention	Second-intervention	Pre-intervention	First-intervention	Second-intervention
Total number of prescriptions	1,532,336	2,450,093	15,499,762	1,503,938	2,123,509	6,254,171
Number and proportion of antibiotic prescriptions	196,139 (12.8)	264,610 (10.8)	325,495 (2.1)	97,756 (6.5)	121,040 (5.7)	256,421 (4.1)
Antimicrobials according to grade, *N* (%)						
Non-restricted antimicrobials	137,046 (9.0)	185,648 (7.6)	230,066 (1.5)	36,966 (2.5)	47,694 (2.3)	98,965 (1.6)
Restricted antimicrobials	57,243 (3.8)	75,461 (3.1)	95,386 (0.6)	50,692 (3.4)	60,111 (2.8)	126,873 (2.0)
Special-grade antimicrobials	1838 (0.1)	3456 (0.1)	29 (0.0)	10,098 (0.7)	13,231 (0.6)	30,581 (0.5)
Antimicrobials according to class, *N* (%)						
Penicillin	4932 (0.3)	8345 (0.3)	18,841 (0.1)	6983 (0.5)	8391 (0.4)	8825 (0.1)
Penicillin preparation	47,978 (3.1)	60,286 (2.5)	71,151 (0.6)	25,223 (1.7)	23,298 (1.1)	32,716 (0.5)
Cephalosporin	105,385 (7.0)	148,592 (6.1)	185,412 (1.2)	48,741 (3.3)	69,324 (3.3)	164,767 (2.7)
Other beta-lactam	1849 (0.1)	1776 (0.1)	7 (0.0)	2474 (0.2)	2238 (0.1)	0 (0.0)
Aminoglycoside	24 (0.0)	38 (0.0)	28 (0.0)	371 (0.0)	408 (0.0)	1300 (0.0)
Carbapenems	3 (0.0)	6 (0.0)	6 (0.0)	4584 (0.3)	7024 (0.3)	20,963 (0.3)
Glycopeptide	0 (0.0)	2 (0.0)	0 (0.0)	1277 (0.1)	1757 (0.1)	6279 (0.1)
Macrolide	34,390 (2.3)	42,405 (1.7)	44,852 (0.3)	5284 (0.4)	4942 (0.2)	13,715 (0.2)
Other antibiotics	1566 (0.2)	3115 (0.1)	5184 (0.0)	2819 (0.2)	3654 (0.2)	7854 (0.1)
Number of inpatients who took antibiotics (%)	–	–	–	7562 (40.0)	9247 (33.9)	20,662 (22.9)
Total expenditure on antibacterial (Yuan)	8,759,640	12,228,653	17,946,806	9,127,932	11,075,074	21,135,351
Proportion of expenditure on antibacterial (%)	17.1	14.0	6.0	28.8	23.2	14.0

The top 10 antibiotics prescribed during three periods are shown in Table S3 in Supplementary Material. For outpatients, although there was a significant decrease in the rate of the No. 1 antibiotic used, the highest rate of amoxicillin and clavulanic acid prescription was observed throughout three periods. In addition, during both the first-intervention period and the second-intervention period, new rankings of the top 10 antibiotics showed a significant decline in the third-generation cephalosporins, such as Cefixime (No. 4 in the pre-intervention, No. 8 after first-intervention, and excluded from the top 10 after second-intervention). For inpatients, a significant drop was observed in the rankings of “Piperacillin and enzyme inhibitor” used (No. 1 → No. 3 → No. 8) after the implementation of interventions. Conversely, the rankings of the second-generation cephalosporins increased obviously (Table S3 in Supplementary Material).

### Changes in the Proportion of Antibiotic Prescriptions among Three Distinct Periods

#### Outpatient-Specific Analysis

**Figure 2:**
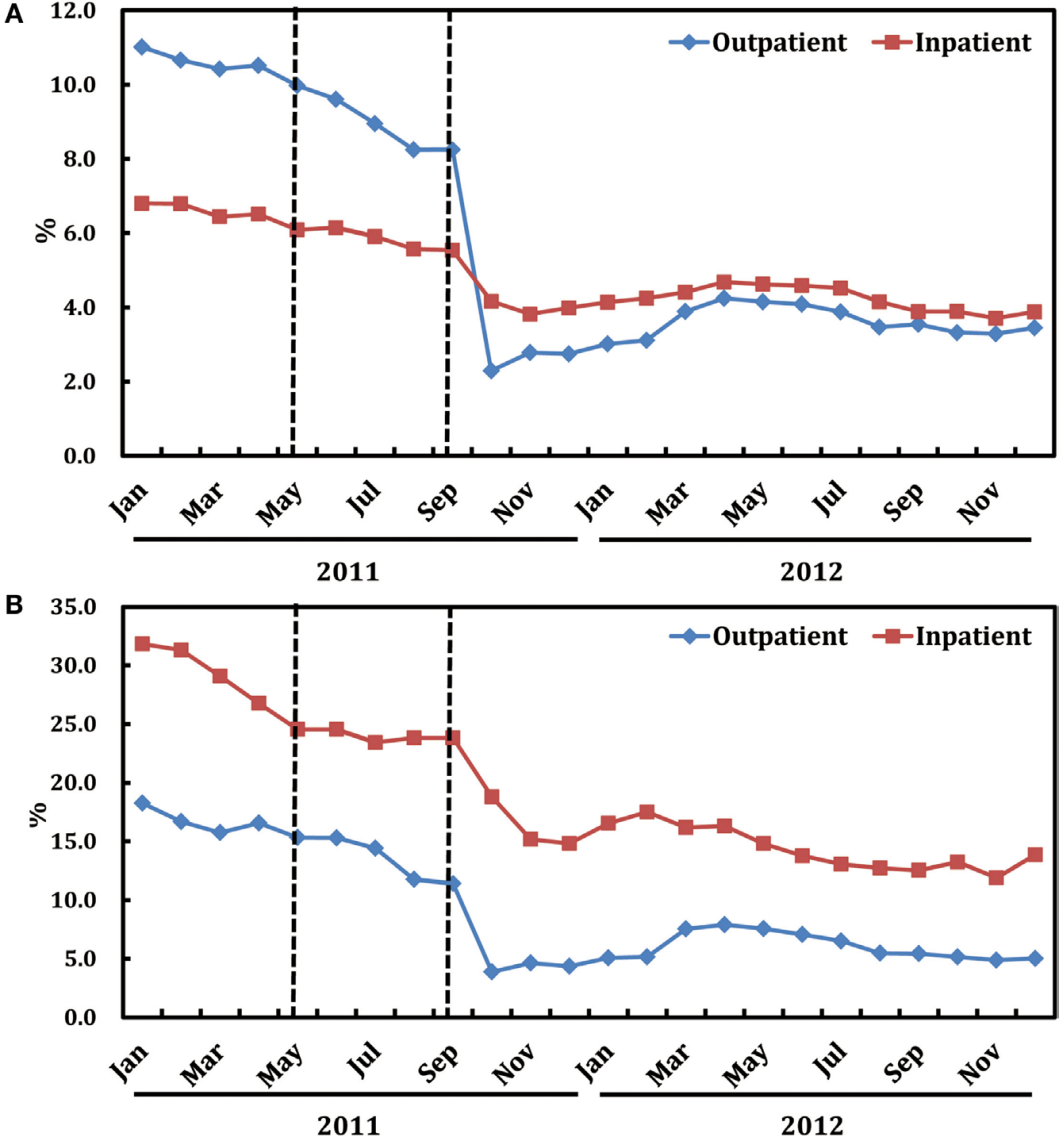
**Trends in the proportion of antibiotic prescriptions (A) and that of expenditure on antibiotics (B) by month in both ambulatory and inpatient settings**.

As shown in Figure 2A, after the release of the ASPs in May 2011, there is a notable decline in the monthly proportion of prescriptions containing antibiotics for outpatients, particularly since the implementation of the financially punished audit and feedback in September 2011. Statistically speaking, proportion of antibiotic prescriptions for outpatients dropped immediately after adding the financially punished audit and feedback to the prior authorization (coefficient: −6.269, *P* < 0.001, reduction: 59.4%) (Table 2). No significant downtrends were identified within pre-intervention period (coefficient: −0.023, *P* = 0.947) and first-intervention period (coefficient: −0.200, *P* = 0.392) (Table 2).

**Table 2:** **Changes in the monthly proportion of antibiotic prescriptions and expenditure on antibiotics in ambulatory setting**.

Indicators	**β**_0_	**β**_1_	*P*_1_	**β**_2_	*P*_2_	**β**_3_	*P*_3_	**β**_4_	*P*_4_	**β**_5_	*P*_5_	*R*^2^
All antibiotics	11.189 (0.708)	−0.023 (0.343)	0.947	−0.418 (0.358)	0.259	−0.200 (0.227)	0.392	−6.269 (0.321)	**<0.001**	0.313 (0.197)	0.132	0.994
Antibiotics according to grade												
Non-restricted	6.929 (0.321)	−0.376 (0.149)	**0.023**	−0.550 (0.323)	0.108	0.164 (0.111)	0.158	−4.127 (0.264)	**<0.001**	0.280 (0.094)	**0.001**	0.992
Restricted	3.909 (0.427)	0.111 (0.203)	0.594	0.023 (0.331)	0.946	−0.242 (0.144)	0.112	−1.357 (0.240)	**<0.001**	0.130 (0.115)	0.275	0.967
Special-grade	0.091 (0.030)	−0.044 (0.013)	**0.004**	0.132 (0.032)	**0.001**	0.003 (0.011)	0.792	−0.031 (0.023)	0.185	0.041 (0.008)	**<0.001**	0.885
Antimicrobials according to class^a^												
Penicillin	0.121 (0.060)	−0.056 (0.027)	**0.056**	−0.017 (0.032)	0.601	0.052 (0.018)	**0.010**	−0.118 (0.029)	**0.001**	0.004 (0.017)	0.823	0.860
Penicillin preparation	3.117 (0.174)	−0.181 (0.061)	**0.009**	−1.266 (0.132)	**<0.001**	0.095 (0.049)	0.070	−0.051 (0.192)	0.793	0.096 (0.078)	0.237	0.987
Cephalosporin	5.862 (0.456)	−0.021 (0.218)	0.926	−0.101 (0.251)	0.693	−0.069 (0.144)	0.639	−3.518 (0.243)	**<0.001**	0.135 (0.128)	0.308	0.990
Other beta-lactam	0.193 (0.008)	0.014 (0.002)	**<0.001**	0.045 (0.009)	**<0.001**	0.020 (0.003)	**<0.001**	−0.034 (0.003)	**<0.001**	−0.035 (0.006)	**<0.001**	0.645
Macrolide	1.513 (0.046)	−0.231 (0.017)	**<0.001**	−0.253 (0.049)	**<0.001**	0.122 (0.017)	**<0.001**	−0.889 (0.026)	**<0.001**	0.128 (0.009)	**<0.001**	0.996
Other antibiotics	0.198 (0.015)	−0.041 (0.005)	**<0.001**	−0.065 (0.012)	**<0.001**	−0.001 (0.004)	0.392	0.070 (0.015)	**<0.001**	0.043 (0.007)	**<0.001**	0.871
Expenditure on antibiotics	18.784 (1.878)	0.145 (0.932)	0.878	−1.703 (1.178)	0.168	−0.555 (0.628)	0.390	−7.777 (0.996)	**<0.001**	0.480 (0.498)	0.349	0.977

*β_0_-intercept, β_1_-trend during pre-intervention, β_2_-immediately changes following the first-intervention, β_3_-trends during the first-intervention period, β_4_-immediately changes following the second-intervention, β_5_-trends during the second-intervention period*.

*^a^The proportions of aminoglycoside and carbapenems were excluded from analysis due to too small. Bold font-Statistically significant results (P < 0.05)*.

In an antibiotic grade-specific analysis, adding of the financially punished audit and feedback led to an immediately sharp decrease in the prescriptions of both non-restricted (*P* < 0.001) and restricted (*P* < 0.001) antibiotics for outpatients (Table 2). According to the class of antibiotics, stratified analyses showed that the prescriptions of penicillin (*P* = 0.001), cephalosporins (*P* < 0.001), other beta-lactam (*P* < 0.001), and macrolide (*P* < 0.001) for outpatients were immediately reduced to a much greater extent after adding financially punished audit and feedback than after the prior authorization alone (Table 2).

#### Inpatient-Specific Analysis

Figure 2A also illustrates the trends in monthly proportions of prescriptions containing antibiotics for inpatients from January, 2011 to December, 2012. Proportion of antibiotic prescriptions for inpatients dropped significantly (coefficient: −1.235, *P* < 0.001, reduction: 19.8%), immediately after the introduction of financially punished audit and feedback (Table 3). Furthermore, there was no significant downtrends before the financially punished audit and feedback, including the single prior authorization intervention period (coefficient: −0.058, *P* = 0.667) (Table 3). The percentage of monthly antibiotic users of hospitalized children was also decreased (coefficient: −9.124, *P* < 0.001, reduction: 15.5%) immediately after the combination of financially punished audit and feedback. Although the subsequent percentage significantly increased during the second-intervention period, the magnitude (3.478 vs. −9.124) was much lower than that of the decrease resulted from the ASPs change (Table 3).

**Table 3:** **Changes in the monthly proportion of antibiotic use and expenditure on antibiotics in inpatient setting**.

Indicators	**β**_0_	**β**_1_	*P*_1_	**β**_2_	*P*_2_	**β**_3_	*P*_3_	**β**_4_	*P*_4_	**β**_5_	*P*_5_	*R*^2^
All antibiotics	6.807 (0.399)	−0.050 (0.193)	0.798	−0.461 (0.245)	0.078	−0.058 (0.132)	0.667	−1.235 (0.216)	**<0.001**	0.089 (0.107)	0.413	0.977
Antibiotics according to grade												
Non-restricted	2.580 (0.123)	−0.026 (0.056)	0.652	−0.026 (0.130)	0.847	−0.025 (0.045)	0.582	−0.561 (0.091)	**<0.001**	0.047 (0.032)	0.156	0.969
Restricted	3.529 (0.367)	0.038 (0.191)	0.843	−0.374 (0.145)	**0.020**	−0.055 (0.125)	0.667	−0.623 (0.115)	**<0.001**	−0.001 (0.100)	0.994	0.975
Special-grade	0.670 (0.052)	−0.034 (0.019)	0.095	0.077 (0.056)	0.184	0.001 (0.020)	0.950	−0.162 (0.030)	**<0.001**	0.041 (0.011)	**0.001**	0.845
Antimicrobials according to class												
Penicillin	0.470 (0.036)	−0.030 (0.017)	0.104	0.027 (0.025)	0.304	−0.001 (0.013)	0.922	−0.093 (0.021)	**0.001**	0.019 (0.008)	**0.030**	0.988
Penicillin preparation	2.216 (0.156)	0.092 (0.076)	0.245	−0.051 (0.128)	0.696	−0.190 (0.054)	**0.003**	−0.151 (0.109)	0.187	0.070 (0.042)	0.113	0.976
Cephalosporin	2.842 (0.329)	−0.129 (0.160)	0.432	−0.278 (0.249)	0.280	0.173 (0.110)	0.135	−0.876 (0.258)	**0.004**	−0.035 (0.092)	0.704	0.842
Other beta-lactam	0.177 (0.011)	−0.032 (0.004)	**<0.001**	0.092 (0.011)	**<0.001**	−0.008 (0.004)	0.057	−0.036 (0.006)	**<0.001**	0.040 (0.002)	**<0.001**	0.982
Aminoglycoside	0.022 (0.006)	−0.002 (0.003)	0.413	−0.004 (0.006)	0.531	0.001 (0.002)	0.577	0.001 (0.004)	0.805	0.001 (0.002)	0.433	0.200
Carbapenems	0.356 (0.044)	0.049 (0.017)	**0.010**	−0.025 (0.048)	0.607	−0.019 (0.017)	0.273	−0.131 (0.027)	**<0.001**	−0.022 (0.009)	**0.037**	0.595
Glycopeptide	0.068 (0.022)	−0.008 (0.010)	0.439	−0.008 (0.024)	0.737	0.007 (0.008)	0.402	−0.001 (0.017)	0.970	0.003 (0.006)	0.570	0.378
Macrolide	0.392 (0.027)	−0.000 (0.012)	0.976	−0.063 (0.029)	**0.044**	−0.013 (0.010)	0.205	0.004 (0.021)	0.849	0.014 (0.007)	0.059	0.880
Other antibiotics	0.265 (0.036)	0.015 (0.018)	0.418	0.071 (0.041)	0.108	−0.029 (0.013)	**0.047**	−0.037 (0.028)	0.210	0.017 (0.010)	0.109	0.789
Inpatients taking antibiotics	59.428 (2.124)	−2.898 (1.015)	**0.012**	2.916 (2.110)	0.186	−0.391 (0.763)	0.616	−9.124 (1.632)	**<0.001**	3.478 (0.570)	**<0.001**	0.982
Expenditure on antibiotics	33.670 (1.579)	1.080 (0.729)	0.158	−1.838 (1.574)	0.260	−1.603 (0.572)	**0.013**	−4.933 (1.126)	**0.001**	0.143 (0.397)	0.723	0.981

*β_0_-intercept, β_1_-trend during pre-intervention, β_2_-immediately changes following the first-intervention, β_3_-trends during the first-intervention period, β_4_-immediately changes following the second-intervention, β_5_-trends during the second-intervention period. Bold font-Statistically significant results (P < 0.05)*.

In subgroup analyses, implementation of the financially punished audit and feedback led to sharp decreases in all three grades of antibiotics in inpatients (all *P* < 0.001) (Table 3). Further stratified analyses of antibiotic kinds demonstrated that combined ASPs could immediately reduce the prescriptions of penicillin (*P* = 0.001), cephalosporins (*P* = 0.004), other beta-lactam (*P* < 0.001), and carbapenems (*P* < 0.001) and continue to play this role for carbapenems (*P* = 0.037) in the subsequent period (Table 3).

### Changes in the Proportion of Expenditure on Antibiotics Relative to All Medications

As shown in Figure 2B, after the release of the financially punished audit and feedback in September, 2011, there is a notable decline in the proportions of expenditure on antibiotics relative to all medications in both outpatient and inpatient wards.

#### Outpatient-Specific Analysis

In the periods before financially punished audit and feedback implementation, even in the first-intervention period during which prior authorization had been started, there were no significant downtrends of the proportions of expenditure on antibiotics relative to all medications (coefficient = 0.145 and *P* = 0.932 for pre-intervention period; coefficient = 0.555 and *P* = 0.390 for the first-intervention period). After the financially punished audit and feedback was implemented, the proportions of expenditure on antibiotics experienced an immediate decline (coefficient: −7.777, *P* < 0.001, reduction: 46.7%) (Table 2).

#### Inpatient-Specific Analysis

In the periods after the prior authorization alone and before the implementation of financially punished audit and feedback, the proportion of expenditure on antibiotics was slowly reduced (coefficient: −1.603, *P* = 0.013). However, after the financially punished audit and feedback was implemented, the proportion of expenditure on antibiotics immediately declined by 16.3% (coefficient: −4.933, *P* = 0.001) (Table 3). The equations should be inserted in editable format from the equation editor.

## Discussion

Medically inappropriate, ineffective, and economically inefficient use of antibiotics is commonly observed in the health-care units throughout the countries of all income levels, especially in the developing countries (18). As China emerging as the largest developing country and the world’s largest producer and user of antibiotics (19), policy makers are under pressure to control inappropriate use of antibiotic without adversely affecting the quality of care. Previous literature supports all three methods (prior authorization, audit and feedback, and pay for performance) as being effective strategies to decrease antimicrobial exposure, decrease costs, and improve clinical outcomes (20, 21). However, because these three ASP methods have not been compared or combined, the most effective approach remains unclear. This is the first study to evaluate whether combining three methods could result in more influences on antibiotic use in pediatrics than one method alone.

After release of the prior authorization alone in May 2011, hospital-wide overall antibiotic use slowly started to decrease. However, these changes were not statistically significant until the combination with financially punished audit and feedback in September 2011. Compared to patterns before combining, the proportions of prescriptions of the overall antibiotic use declined nearly 60 and 20% for outpatients and inpatients, respectively. Correspondingly, the proportion of expenditure on antibiotics relative to all medications decreased approximately 47 and 16% for outpatients and inpatients, respectively. These findings suggest that financially punished audit and feedback is capable of catalyzing improvement in efforts on appropriate use of antibiotics in pediatric hospital that is already engaged in prior authorization.

Generally, our findings are in line with and build on the existing evidence based on the previous studies that examined the effect of pediatric ASPs. Hersh et al. reported an average monthly decline in days of therapy/1000 patient days of 5.7% after implementing an ASP compared with control hospitals within the Pediatric Health Information System network (22). Di Pentima et al. reported a significant impact on reducing antimicrobial use after implementing an ASP, with the reductions of approximately 21 and 50% in antibiotic doses administered per 1000 patient days of targeted and non-targeted antimicrobial, respectively (23). In another study, an ASP was associated with a $370,069 reduction in projected annual cost related to restricted antimicrobial use (24). Regrettably, direct comparisons with our study are not available because the metrics used in these studies were not consistent. But, all these findings suggest that formalized ASPs in children’s hospitals are effective for improving antibiotic prescribing.

However, our study did not observe a significant decrease in all antimicrobial use in both ambulatory and inpatient clinical settings after the implementation of prior authorization alone, based on the recommendation made by the “Clinical Application of Antibiotics Special Rectification Activities in 2011.” Although other explanations may limit the interpretation of this finding, our results implicated that the national special rectification activity without an effective antimicrobials stewardship cannot efficiently decrease the inappropriate and indiscriminate use of antibiotics. It may potentially be able to explain a given observed phenomenon that antibiotic use still remains at a higher level, and antibiotic resistance remains a challenge in China, despite sets of guidelines and regulations had been released during the past decade (25).

Understanding of which specific stewardship strategy is the most effective one, especially the intervention requiring lower levels of financial support, is particularly important (26). Unfortunately, so far, there is a lack of conclusive studies to draw definite conclusions of efficacy of different ASP interventions. As one of the most widely advocated strategies, little is known about the effect of prospective audit and feedback on reducing antibiotic prescribing in children’s hospital. In one well-documented case, compared with the antibiotic use of the control group, a monthly decline in all antibiotics of 7% (*P* = 0.05) and 8% (*P* = 0.05) was observed for days of therapy and length of therapy per 1000 patient days, respectively (27). Hospitals with audit and feedback ASPs did reduce antibiotic use to a greater degree than those without. However, this was not universal. For example, according to a study run by Mehta et al. (8), after the introduction of prospective audit with feedback, both total antimicrobial use (*P* < 0.001) and broad-spectrum anti-Gram-negative antimicrobial use (*P* < 0.001) increased significantly.

As another widely advocated strategy, even less is known about the effects of financial incentives/penalties for prescribers. According to the latest available updates of a systematic review in 2015 (28), (i) pharmaceutical budgets may lead to a modest reduction in drug use (median relative change −2.8%; low-certainty evidence); (ii) effects of pay for performance policies on drug use and health outcomes are uncertain, and effects on drug costs and health-care utilization have not been measured; (iii) effects of the reimbursement rate reduction policy on drug use and drug costs are uncertain, and no included study assessed the effects of this policy on health-care utilization or health outcomes; and (iv) effects of financially punished policy were not reported at all.

Although both favorable clinical and economic outcomes were observed in the post-intervention phase, several limitations associated with this study should be acknowledged. First, process measures, such as the proportion of antibiotic prescriptions and expenditure on antibiotics, are considered inadequate to evaluate ASP interventions, because such outcomes do not demonstrate direct clinical benefits (29). For example, one important focus of ASPs is to guide clinicians from broader- to narrower-spectrum antibiotics. However, switch from one agent to another for de-escalation would not be demonstrated by the proportion of antibiotic prescriptions and expenditure metrics. Additionally, many other important outcomes related to ASPs, including changes in days of therapy per 1000 patient days, rates of multidrug-resistant organisms and *Clostridium difficile* infection, conservable days of therapy, and unplanned hospital readmission within 30 days after discharge from the hospital, were not measured in this study (30).

Second, this study is in essence a description of what happened in a pediatric hospital after introducing ASPs. It is just an uncontrolled, observational study without simultaneous control group. Thus, we should note all the limitations of observational studies, and this study design does not permit clear determination of causation in the changes observed. Third, patient adherence to treatment regimens was not monitored, so that prescribing data may not accurately represent actual antibacterial use, particularly for outpatients (31). Fourth, the influence of factors other than the change in ASP cannot be fully excluded. For instance, the decreased use of certain types of antibiotics before the intervention suggests that external factors, such as seasonal variation that January is a peak season of some pediatric infectious diseases (32), may have lead to a downtrend. Fifth, time duration is likely too short to avoid an additive effect. For example, interventions in the hospital level take time to “diffuse” through and 3 months of the first-intervention period is too short to be able to tease out the individual effects of each intervention introduced. To increase the validity of the results, future research should include several years of data before and, ideally, after implementation of similar ordinances and should use statistical methods that control for secular trends and random effects. Finally, the success of interventions depends on the specific prescribing behaviors and specific barriers to behavior change in each setting. Thus, the generalizability of our findings to non-freestanding children’s hospitals is uncertain.

In conclusion, even with all the limitations of any observational study, this study observed a significant decline in antibiotic use and corresponding expenditure in both ambulatory and inpatient clinical settings after the inclusion of financially punished audit and feedback to an ASP based on prior authorization alone. This implies that the combination of more than one core strategies (prior authorization, audit and feedback, and pay for performance) will be more effective than one strategy alone. We are initiating another research project to determine whether financial incentives or the restructuring of payment models can stimulate more meaningful improvements.

## Author Contributions

Study concept and design: SG, HL, and HY; analysis and interpretation of data: XQ, SS, YL, and HL; acquisition of data: YS, XS, ML, RL, and QW; drafting of the manuscript: SG, HX, and HY; administrative, technical, and material support: YH, YC, LH, LZ, WZ, JM, LL, and YX; critical revision of the manuscript for important intellectual content: HX, SG, and HL; and all authors critically read the manuscript, revised it, and approved the final version.

## Conflict of Interest Statement

The research was conducted in the absence of any commercial or financial relationships that could be construed as a potential conflict of interest.

## Abbreviations

GWCMC, Guangzhou Women and Children’s Medical Center.
