# Supplementary material for: Effect of Financially Punished Audit and Feedback in a Pediatric Setting in China, within an Antimicrobial Stewardship Program, and as Part of an International Accreditation Process
Source: Front Public Health. 2016 May 18;4:99. doi: 10.3389/fpubh.2016.00099 (PMC4870519; doi:10.3389/fpubh.2016.00099)
Supplement: Supplementary file 1 [file table_1.docx]

Supplementary Material

Financially punished audit & feedback making antimicrobial stewardship program in pediatric actionable during the journey to joint commission international accreditation

**Sitang Gong, Xiu Qiu, Yanyan Song, Xiu Sun, Yanling He, Yilu Chen, Minqing Li, Rui Luo, Liya He, Qing Wei, Songying Shen, Yu Liu, Lian Zhang, Wei Zhou, Ping Huang, Jianning Mai, Li Liu, Yi Xu, Huiying Liang, Huimin Xia^*^**

*** Correspondence: Huimin Xia**: [huimin.xia876001@gmail.com](mailto:huimin.xia876001@gmail.com)

# Supplementary Table S1

| **Supplementary Table S1.** Clinical indications for the rational use of antibiotics in children | |
| --- | --- |
| **ID** | **Indications for antibiotic treatment** |
| **When not to treat with an antibiotic** | |
| 1 | Getting fever less than 3 days without abnormal level of white blood cell count and C-reactive protein or aberrant classification of white blood cell; |
| 2 | Getting fever less than 5 days without evidence of infection, e.g. normal level of white blood cell count and C-reactive protein or classification of white blood cell, negative blood culture or pathogenic examination. |
| **When to treat with antibiotics** | |
| 1 | Infectious diseases caused by the following pathogens, including bacteria, fungi, mycobacterium tuberculosis, non-tuberculous mycobacteria, mycoplasma, chlamydia, spirochetes, rickettsia and some protozoa and other pathogenic microorganisms, based on symptoms, signs and positive pathology examination in blood, urine or stool; |
| 2 | The preventive use of antibiotics in patients who had defects or poor immune function, was using of immunosuppressive agents, had severe malnutrition, premature birth, or had severe basic diseases, refer to the guideline of principle clinical use of antibiotics and principles of prophylactic antibiotics published by Ministry of Health of China; |
| 3 | Usage of antibiotics in patients with serious disease condition without certain pathogen, which is approved by two associate chief physicians; |
| 4 | Other considerations requiring use of antimicrobial drugs, and provided evidence of infection or other basis of pathogenic bacteria use; |
| 5 | High risk in antibiotic usage, but it is approved by the antimicrobial stewardship program team; |
| 6 | The utilization of prophylactic antibiotics in patients undergoing Type-I incision operation, and approved by chief of the clinical medical group. |
| **Note:** Although the summary describes recommended courses of intervention, it is not intended as a substitute for the advice of a physician or other knowledgeable health care professionals. | |
