# Supplementary material for: Effect of Financially Punished Audit and Feedback in a Pediatric Setting in China, within an Antimicrobial Stewardship Program, and as Part of an International Accreditation Process
Source: Front Public Health. 2016 May 18;4:99. doi: 10.3389/fpubh.2016.00099 (PMC4870519; doi:10.3389/fpubh.2016.00099)
Supplement: Supplementary file 2 [file table_2.docx]

Supplementary Material

Financially punished audit & feedback making antimicrobial stewardship program in pediatric actionable during the journey to joint commission international accreditation

**Sitang Gong, Xiu Qiu, Yanyan Song, Xiu Sun, Yanling He, Yilu Chen, Minqing Li, Rui Luo, Liya He, Qing Wei, Songying Shen, Yu Liu, Lian Zhang, Wei Zhou, Ping Huang, Jianning Mai, Li Liu, Yi Xu, Huiying Liang, Huimin Xia^*^**

*** Correspondence: Huimin Xia**: [huimin.xia876001@gmail.com](mailto:huimin.xia876001@gmail.com)

# Supplementary Table S2

| **Supplementary Table S2.** Formulary adjustment & classification management of the antibiotics used in the Guangzhou Women and Children’s Medical Center during 2011-2012 | | | | |
| --- | --- | --- | --- | --- |
| **Category** | **Non-restricted** | **Restricted** | | **Special-grade** |
| **Penicillin** | Amoxicillin(J01CA04)/Ampicillin(J01CA01)/ Oxacillin(J01CF04)/Penicillin G benzathine (J01CE08)/Nafcillin(J01CF06)/ Piperacillin (J01CA12)/Penicillin(J01CA12) | Sulbenicillin(J01CA16)/Mezlocillin(J01CA10) | | — |
| **Beta-lactam or**  **beta-lactamase inhibitor combinations** | Amoxicillin and clavulanic acid(J01CR02) | Amoxicillin and flucloxacillin(J01CR)/Amoxicillin and sulbac tam (J01CR05)/Ampicillin and sulbactam (J01CR01) /Mezl ocillin and sulbactam(J01CR)/Piperacillin and sulbactam (J 01CR05)/ Piperacillin and tazobactam (J01CR05)/ Latamo xef(J01DD07) /Cefmetazole (J01DC09) /Cefminox(J01DI)/ Cefoxitin(J01DC01) | — | |
| **Cephalosporin** | Cephalexin(J01DB01)/Cefprozil(J01DC10)/ Cefuroxime(J01DC02)/Cefaclor(J01DC04)/ Cefradine (J01DB09)/Cefadroxil(J01DB05)/ Ceftriaxone(J01DD04)/Cefazolin(J01DB04) | Cefpodoxime(J01DD13)/Cefdinir(J01DD15)/Cefodizime(J01DD09)/Cefixime(J01DD08)/Cefathiamidine(J01DB)/Cefamandole(J01DC03)/Cefoperazoneandsulbactam(J01DD12)/Cefoperazoneandtazobactam(J01DD62)/Ceftriaxoneandtazobactam(J01DD54)/Cefotaxime(J01DD01)/Ceftazidime(J01DD02)/Cefetamet(J01DD10)/Cefterampivoxil(J01DD)/Cefotiam(J01DC07)/Ceftezole(J01DB12)/Ceftizoxime(J01DD07) | | Cefepime(J01DE01)  Cefmenoxime(J01DD05) |
| **Aminoglycoside** | Amikacin (J01GB06)/Gentamicin(J01GB03) | Tobramycin(J01GB01) | | — |
| **Carbapenems** | — | — | | Meropenem(J01DH02)  Panipenem and betamipron(J01DH55)  Imipenem and cilastatin(J01DH51) |
| **Glycopeptide** | — | — | | Vancomycin(J01XA01) |
| **Macrolide** | Azithromycin(Oral, J01FA10)/ Erythromycin (J01FA01)/Erythromycin ethylsuccinate (J01F A10)/Josamycin(J01FA07)/Clarithromycin(J01FA09)/Clindamycin(J01FA09)/Roxithromycin (J01FA06)/Erythromycin estolate(J01FA02) | Azithromycin(Injection , J01FA10) | | — |
| **Others** | Doxycycline(J01AA02) | Fusidic acid(J01XC01)/Chloramphenicol(J01BA01) | | Linezolid(J01XX08) |
