# Supplementary material for: Effect of Financially Punished Audit and Feedback in a Pediatric Setting in China, within an Antimicrobial Stewardship Program, and as Part of an International Accreditation Process
Source: Front Public Health. 2016 May 18;4:99. doi: 10.3389/fpubh.2016.00099 (PMC4870519; doi:10.3389/fpubh.2016.00099)
Supplement: Supplementary file 3 [file table_3.docx]

Supplementary Material

Financially punished audit & feedback making antimicrobial stewardship program in pediatric actionable during the journey to joint commission international accreditation

**Sitang Gong, Xiu Qiu, Yanyan Song, Xiu Sun, Yanling He, Yilu Chen, Minqing Li, Rui Luo, Liya He, Qing Wei, Songying Shen, Yu Liu, Lian Zhang, Wei Zhou, Ping Huang, Jianning Mai, Li Liu, Yi Xu, Huiying Liang, Huimin Xia^*^**

*** Correspondence: Huimin Xia**: [huimin.xia876001@gmail.com](mailto:huimin.xia876001@gmail.com)

# Supplementary Table 3

| **Supplementary Table S3.** The topic ten uses of antibiotics and correspoding percentage of prescriptions during the pre-NSRA, pre-ASP and ASP for both outpatients and inpatients | | | | | | | | | | | | | | | | |
| --- | --- | --- | --- | --- | --- | --- | --- | --- | --- | --- | --- | --- | --- | --- | --- | --- |
| **Outpatients** | | | | | | | |  | **Inpatients** | | | | | | | |
| **First phase** | |  | **Second phase** | |  | **Third phase** | |  | **First phase** | |  | **Second phase** | |  | **Third phase** | |
| **Antibiotic**  **(ATC codes)** | **n(%)** | **Antibiotic**  **(ATC codes)** | | **n(%)** | **Antibiotic**  **(ATC codes)** | | **n(%)** | **Antibiotic**  **(ATC codes)** | | **n(%)** | **Antibiotic**  **(ATC codes)** | | **n(%)** | **Antibiotic**  **(ATC codes)** | | **n(%)** |
| Amoxicillin and clavulanic acid (J01CR02) | 39952  (2.62) | Amoxicillin and clavulanic acid (J01CR02) | | 49628  (2.03) | Amoxicillin and clavulanic acid (J01CR02) | | 60975  (0.39) | Piperacillin and enzyme inhibitor (J01CR05) | | 10651  (0.71) | Cefamandole (J01DC03) | | 15617  (0.74) | Cefamandole (J01DC03) | | 61022  (0.98) |
| Clarithromycin (J01FA09) | 16707  (1.09) | Cefprozil (J01DC10) | | 24384  (1.00) | Cefamandole (J01DC03) | | 37875  (0.24) | Amoxicillin and clavulanic acid (J01CR02) | | 10378  (0.69) | Cefuroxime (J01DC02) | | 13671  (0.65) | Cefoperazone (J01DD12) | | 27288  (0.44) |
| Cefprozil (J01DC10) | 16264  (1.07) | Clarithromycin (J01FA09) | | 23438  (0.96) | Cefuroxime (J01DC02) | | 26182  (0.17) | Cefuroxime (J01DC02) | | 7031  (0.47) | Piperacillin and enzyme inhibitor (J01CR05) | | 11335  (0.54) | Cefuroxime (J01DC02) | | 25885  (0.42) |
| Cefixime (J01DD08) | 9006  (0.59) | Cefamandole (J01DC03) | | 16250  (0.66) | Clarithromycin (J01FA09) | | 24530  (0.16) | Cefamandole (J01DC03) | | 6774  (0.45) | Amoxicillin and clavulanic acid (J01CR02) | | 8490  (0.40) | Amoxicillin and clavulanic acid (J01CR02) | | 15808  (0.25) |
| Azithromycin (J01FA10) | 8395  (0.55) | Cefuroxime (J01DC02) | | 11905  (0.49) | Cefprozil (J01DC10) | | 20761  (0.13) | Cefoperazone (J01DD12) | | 6324  (0.42) | Cefoperazone (J01DD12) | | 8289  (0.40) | Ceftriaxone (J01DD04) | | 15269  (0.25) |
| Cefaclor (J01DC04) | 7635  (0.50) | Azithromycin (J01FA10) | | 10272  (0.42) | Azithromycin (J01FA10) | | 20303  (0.13) | Cefodizime  (J01DD09) | | 5039  (0.34) | Benzylpenicillin (J01CE01) | | 5079  (0.24) | Meropenem  (J01DH02) | | 13853  (0.22) |
| Cefuroxime (J01DC02) | 7323  (0.48) | Cefazolin (J01DB04) | | 9525  (0.39) | Cefazolin (J01DB04) | | 17674  (0.11) | Benzylpenicillin (J01CE01) | | 4480  (0.30) | Cefazolin  (J01DB04) | | 4566  (0.22) | Cefazolin  (J01DB04) | | 13143  (0.21) |
| Cefamandole (J01DC03) | 7027  (0.46) | Cefixime (J01DD08) | | 9015  (0.37) | Mezlocillin  (J01CA10) | | 16529  (0.11) | Ceftriaxone (J01DD04) | | 3937  (0.26) | Cefaclor  (J01DC04) | | 4465  (0.21) | Piperacillin and enzyme inhibitor (J01CR05) | | 13082  (0.21) |
| Cefotiam (J01DC07) | 6293  (0.41) | Cefadroxil (J01DB05) | | 8489  (0.35) | Ceftriaxone (J01DD04) | | 12538  (0.08) | Ceftizoxime (J01DD07) | | 3933  (0.26) | Cefodizime  (J01DD09) | | 4344  (0.21) | Azithromycin (J01FA10) | | 12080  (0.19) |
| Cefdinir (J01DD15) | 6140  (0.40) | Cefaclor (J01DC04) | | 8489  (0.35) | Cefdinir (J01DD15) | | 10907  (0.07) | Amoxicillin and flucloxacillin (J01CR) | | 3402  (0.23) | Meropenem  (J01DH02) | | 4218  (0.20) | Benzylpenicillin (J01CE01) | | 7296  (0.12) |
