# Supplementary material for: Effect of Financially Punished Audit and Feedback in a Pediatric Setting in China, within an Antimicrobial Stewardship Program, and as Part of an International Accreditation Process
Source: Front Public Health. 2016 May 18;4:99. doi: 10.3389/fpubh.2016.00099 (PMC4870519; doi:10.3389/fpubh.2016.00099)
Supplement: Supplementary file 4 [file image_1.pdf]

Supplementary Material

Financially punished audit & feedback making antimicrobial stewardship program in pediatric actionable during the journey to joint commission international accreditation

Sitang Gong, Xiu Qiu, Yanyan Song, Xiu Sun, Yanling He, Yilu Chen, Minqing Li, Rui Luo, Liya He, Qing Wei, Songying Shen, Yu Liu, Lian Zhang, Wei Zhou, Ping Huang, Jianning Mai, Li Liu, Yi Xu, Huiying Liang, Huimin Xia*

*** Correspondence: Huimin Xia**: huimin.xia876001@gmail.com

2 Supplementary Figures

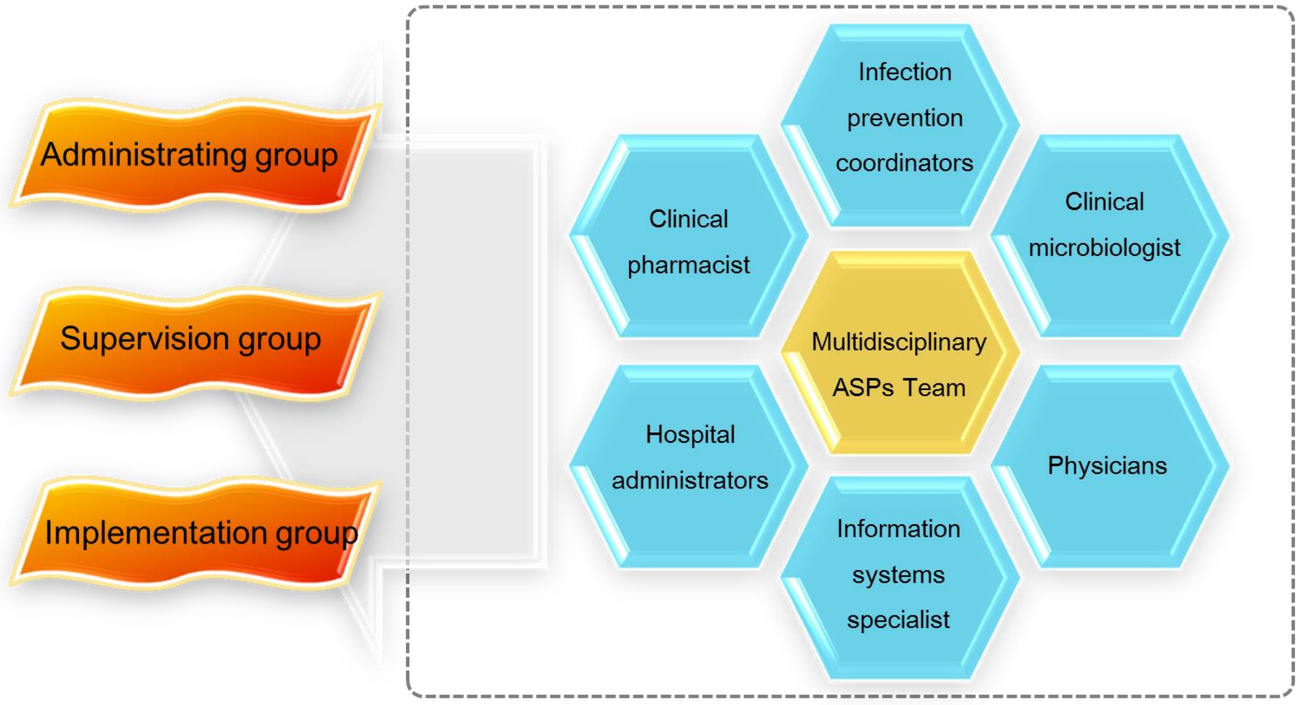

Supplementary Figure S1. Members and structure of the antimicrobial stewardship program (ASP) team.
